# Emerging markers of cancer cachexia and their relationship to sarcopenia

**DOI:** 10.1007/s00432-023-05465-9

**Published:** 2023-10-31

**Authors:** Melanie Lipshitz, J. Visser, R. Anderson, D. G. Nel, T. Smit, H. C. Steel, B. Rapoport

**Affiliations:** 1 Division of Human Nutrition, Stellenbosch University, Stellenbosch, South Africa; 2 Melanie Levy Dietician, 1 Mid Way Road, Glenhazel, Johannesburg, South Africa; 3 Department of Immunology, University of Pretoria, Pretoria, South Africa; 4 Centre for Statistical Consultation, Stellenbosch University, Stellenbosch, South Africa; 5 The Medical Oncology Centre of Rosebank, Johannesburg, South Africa

**Keywords:** Cachexia, Biomarkers, Sarcopenia, Hand grip strength, Skeletal muscle index

## Abstract

**Purpose:**

Emerging biomarkers of cancer cachexia and their roles in sarcopenia and prognosis are poorly understood. Baseline assessments of anthropometrics, sarcopenia, cachexia status and biomarkers of cachexia were measured in patients with advanced cancer and healthy controls. Thereafter, relationships of the biomarkers with cachexia and sarcopenia were explored.

**Methods:**

A prospective case–control design was used, including 40 patients with advanced cancer and 40 gender, age-matched controls. Bioelectrical impedance [skeletal muscle index (SMI)] and hand dynamometry [hand grip strength (HGS)] assessed sarcopenia and a validated tool classified cancer cachexia. Albumin, lymphocyte and platelet counts, haemoglobin, C-reactive protein (CRP), pro-inflammatory cytokines/chemokines and citrullinated histone H3 (H3Cit) were measured.

**Results:**

Patients had significantly lower SMI (6.67 kg/m^2^ versus 7.67 kg/m^2^, *p* =  < 0.01) and HGS (24.42 kg versus 29.62 kg) compared to controls, with 43% being sarcopenic**.** Significant differences were found for albumin, lymphocyte and platelet counts, haemoglobin, CRP, and tumour necrosis factor α (TNFα), (*p* < 0.01). Interleukin (IL)-6 (*p* < 0.04), IL-8 (*p* = 0.02), neutrophil/lymphocyte ratio (NLR), *p* = 0.02, platelet/lymphocyte (PLR) ratio, *p* < 0.01 and systemic immune inflammatory index (SII), *p* < 0.01 differed significantly. No difference was observed for CXC motif chemokine ligand 5 [CXCL5 or epithelial neutrophil-activating peptide 78 (ENA78)] or H3Cit. Albumin and haemoglobin correlated negatively with total protein, skeletal muscle mass and SMI (all *p* < 0.01). The presence of sarcopenia associated significantly with albumin, haemoglobin and CRP.

**Conclusion:**

Significant relationships and differences of haemoglobin, CRP and albumin supports future use of these biomarkers in cancer cachexia. CXCL5 and H3Cit as valuable biomarkers in cancer cachexia remains to be defined.

## Introduction

Cancer cachexia is defined as a multi-factorial and complex metabolic syndrome (Ryan et al. 2016) characterized by an ongoing loss of skeletal muscle mass (with or without functional impairment) that cannot be fully reversed by conventional nutritional support and leads to progressive functional impairment (Srdic et al. 2016).

Cachexia involves many different metabolic pathways and is typified by systemic inflammation, ongoing weight loss and reductions of adipose tissue and skeletal muscle. Metabolically, cachexia does not support anabolism, but rather propagates catabolism and ultimately compromises energy balance (Peixoto et al. 2020). This catabolism manifests not only as depleting skeletal muscle mass but may also affect major organs directly.

The term sarcopenia is typically used to define an age-related decrease in muscle mass that is characterized by a reduction in muscle mass resulting in a loss of strength, functional impairments and disability (Jones et al. 2009). However, cancer cachexia and sarcopenia often overlap, especially in older patients, where cachexia presents with weight loss and sarcopenia presents with the loss of skeletal muscle (Peixoto et al. 2020). Secondary sarcopenia is defined as the lean body mass reduction that occurs as a result of acute and chronic disease states including cancer, infections, chronic organ failure, immobilization, and disability (Suetta 2019). The differentiation of primary and secondary sarcopenia or their respective overlaps may be especially challenging if an elderly sample is being studied. Sarcopenia is accepted as an integral element of cancer cachexia and has been widely accepted to play a pivotal role in clinical assessments, identified by an ongoing reduction of skeletal muscle mass, muscle strength, and physical performance (Srdic et al. 2016).

Advanced cachexia may only become apparent when there is a gross loss of skeletal muscle and when the opportunity for nutritional intervention has passed (Fearon et al. 2013). Optimal responses to nutrition therapy occur when the disease is stable and life expectancy exceeds 3 months (Fiala et al. 2016). Therefore, proper diagnosis and staging of cachexia assists clinicians to ensure goal-directed treatment (Argilés et al. 2019). However, defining cachexia is challenging due to the multitude of methods employed to define cachexia. Pre-cachexia, cachexia, and refractory cachexia classifications defined by a cachexia staging score (CSS) tool (incorporating weight loss, sarcopenia, performance status, appetite and abnormal biochemistry), is optimal for complete and comprehensive cachexia staging (Dev 2018).

The abundance of methods used to measure and define cachexia, makes meaningful comparisons between studies challenging. Less detailed definitions of cachexia use cut-offs of either involuntary weight loss > 10% or a BMI of < 18 kg/m^2^ (Dev 2018). However, recommendations support that when weight loss exceeds 5%, the risk of mortality is greatly increased, necessitating the need for more sensitive criteria, over and above weight loss and BMI alone, to identify patients that are still in the early stages of cachexia (Bruggeman 2016). Broadening cachexia assessments to include questionnaires of anorexia, body composition analysis, markers of inflammation, resting energy expenditure (REE) and physical performance should be integrated to optimize cachexia definitions and to ultimately hinder the progression of malnutrition (Dev 2018). Additionally, factors inclusive of gender, ethnicity, socioeconomic factors and the underlying primary diagnosis should be considered to minimize the challenges in controlling for confounding differences in the assessment of cachexia (Montalvo et al. 2018; Olaechea et al. 2023).

Sarcopenia assessment using either handgrip strength (HGS) or skeletal muscle mass (SMM) plays a pivotal role in the clinical evaluation of cachexia (Srdic et al. 2016), where SMM specifically, is an independent prognosticator of mortality and survival in cancer cachexia (Loumaye and Thissen 2017).

Systemic inflammation, (a hallmark of cancer cachexia) (Aoyagi et al. 2015), is mediated by inflammatory cytokines and underlies the pathogenesis of cachexia (Fearon et al. 2013). Cytokines drive decreased appetite, increased muscle breakdown and energy expenditure (Loumaye and Thissen 2017). Biomarkers that identify cancer cachexia in the early stages may predict progression, and outcomes and guide clinicians to develop early interventions (Mondello et al. 2014). However, clinically effective and reliable biomarkers for cachexia diagnosis are lacking (Ohmori 2019), therefore, ongoing research to determine the ideal biomarker of cancer cachexia is necessary (Loumaye and Thissen 2017). 

Cytokines that have been shown to impact on nutritional status in cachexia include C-reactive protein (CRP), tumour necrosis factor α (TNFα), interleukin (IL)-6 and IL-8 by triggering muscle wasting (Miyamoto et al. 2016) and negatively impacting survival in cancer (Lerner et al. 2015). Furthermore, these biomarkers promote tumour progression, proliferation and survival of malignant cells (Paczek et al. 2020).

Cytokines, as a result of the inflammatory response, are the mediators of the cachexia process: they alter macronutrient metabolism, depress appetite and initiate the acute phase protein (APP) response. Furthermore, the inflammatory cytokines initiate metabolic pathways that increase the release of enzymes that trigger muscle protein turnover (Ryan et al. 2016). Cytokines also play an important role in causing secondary nutrition impact symptoms (S-NIS) including early satiation, constipation, depression and uncontrolled nausea and vomiting (Dev 2018) and may act by driving a systemic suppression of the immune system (Brocco et al. 2019).

Notwithstanding, that APPs and cytokines mediate inflammation peripherally in cancer cachexia, centrally, hypothalamic inflammation results in a stimulation of the hypothalamic–pituitary–adrenal axis, resulting in the release of glucocorticoids. This results in lipolysis and skeletal muscle catabolism, therefore, directly contributing to the metabolic aberrations and compromised nutritional status of cachexia (Peixoto et al. 2020).

Many cancer patients are treated only when a significant amount of weight loss is detected, or when the patients suffer from limitations in their daily living activities and a compromised quality of life. Testing of biomarkers of the cancer cachexia process may, therefore, serve to detect early changes before any clinical manifestations arise, facilitating treatment and, possibly, improving prognosis (Argilés et al. 2019). An improved understanding of the molecular mechanisms and role of these cachectic potentiators in driving cachexia shows promise in discovering new therapeutic targets and to ultimately improve the treatment of cancer cachexia (Miyamoto et al. 2016).

Although TNFα has been accepted as a mediator of cancer cachexia for many years, TNFα inhibitors (both a recombinant fusion protein of TNFα type II receptor which blocks TNFα activity or a recombinant anti-TNFα antibody) have not demonstrated meaningful clinical benefits with respect to reductions in muscle wasting or restorations of lean body mass (Miyamoto et al. 2016). However, tumour necrosis factor receptor-associated factor 6 (TRAF6) which is a TNFα receptor adapter protein that is up-regulated during atrophy and has been found to be over expressed in muscle from gastric cancer patients and when inhibited has shown reductions in skeletal muscle wasting (Porporato 2016).

ALD518, a humanized monoclonal antibody that binds with high affinity to human IL-6, is being refined for the treatment of anaemia, cachexia, and fatigue. A phase I study of nine patients with advanced cancer has reported statistically meaningful differences in hand grip strength and fatigue after ALD518 administration, albeit with a small sample size (Miyamoto et al. 2016) and others have shown a reversal of anorexia, fatigue, and anaemia, but no significant effect on the loss of lean body mass in weight-losing lung cancer patients (Fearon 2012). This is supported in the research done by Ohmori et al. where IL-6 was not found to be correlated to SMI (Ohmori 2019). The clinical significance of IL-6 is well established in predicting survival in advanced cancer, however, the relationship between elevated circulating IL-6 levels and weight loss in cancer patients remains inconsistent in the literature and the association between IL-6 and the low muscularity is poorly researched (Loumaye et al. 2017), supporting the search for reliable markers in this regard.

Hou et al. investigated several cytokines including IL-6, IL-1, TNFα together with IL-8 in patients with advanced pancreatic cancer. Their results indicated that IL-8 levels are critical to the development of cancer cachexia. They found that IL-8 expression was positively correlated to tumour size and also correlated with both increased levels of sarcopenia and weight loss (Hou et al. 2018). Weight loss alone, was used as a defining factor of cachexia but no differences were evaluated or reported with respect to patients categorized as pre-cachexia, cachexia or refractory cachexia. Using a more detailed definition of cancer cachexia may have produced improved accuracy of predicting survival and prognosis using IL-8 as a biomarker.

CXC motif chemokine ligand 5 (CXCL5) and citrullinated histone H3 (H3Cit) are emerging biomarkers whose roles in cancer cachexia are mostly undetermined. CXCL5 is shown to be elevated in various types of malignancies and is associated with metastasis (Hu et al 2018). Cancer-associated inflammation, neutrophil activation, and the release of H3Cit have been found to be linked to cachexia progression, where patients with metastatic spread present with markedly raised H3Cit levels compared to healthy individuals (Thålin et al. 2018). To date, no research has been conducted to determine if there are relationships between CXCL5 and H3Cit to skeletal muscle breakdown and sarcopenia.

There appears to be paucity in the literature on the roles of CXCL5 and H3Cit in cancer cachexia, specifically pertaining to nutrition-related indices. Therefore, a comprehensive cancer cachexia assessment, investigating relationships of emerging and previously investigated biomarkers with nutritional status is warranted (Argilés et al. 2019) to optimise cancer cachexia management. The primary objectives of the current study were to identify relationships between emerging biomarkers of cancer cachexia, sarcopenia status and cachexia status in patients with advanced cancer.

## Methods

### Study population

Forty patients with advanced-stage 4 malignancies (multiple diagnoses, in different stages of chemotherapy treatment) and 40 healthy age and gender-matched controls, using purposive sampling, were included in a prospective case–control design. One-way ANOVA calculations achieved a 90% power (effect size of 0.52). A hypothesis test of equal means defined a significance level of 5%.

All participants were adults older than 18 years. Confounding conditions that excluded eligibility were severe or chronic illnesses of the liver, chronic kidney disease, inflammatory gastrointestinal tract disorders (ulcerative colitis, Crohn’s disease), severe chronic obstructive lung disease, congestive heart failure, insulin-dependent diabetes mellitus, active uncontrolled infection, neuromuscular disorders with hemiplegia, and rheumatoid arthritis affecting the hands, and patients that did not consent to take part in the study. Control participants were volunteers—self-reported to be healthy and not taking any chronic medications pertaining to the relevant exclusions.

### Anthropometric parameters

Anthropometric parameters, including weight and height, were measured, and body mass index (BMI) was calculated. Body composition analysis was assessed using the InBody120 (Gangnam-gu, Seoul, South Korea) that yielded SMM, total body protein and skeletal muscle index (SMI). Sarcopenia was defined by SMI using accepted cut-offs [< 7.23 kg per square meter (kg/m^2^) for males and < 5.67 kg/m^2^ for females] (Chen et al. 2020). Handgrip strength, measured in kg, used the Saehan dynamometer (Saehan Corporation, Gyeongsangam, South Korea). Prescribed guidelines for HGS testing (subjects were asked to perform a maximal contraction for a few seconds using the non-dominant hand) was assessed and sarcopenia defined by cut-offs of < 18 kg for females and < 28 kg for males (Chen et al. 2020). A five-factor cachexia score staging tool [including percentage weight loss in past 6 months; strength, assistance with walking, rise from a chair, climb stairs and falls (SARC-F); Eastern Cooperative Oncology Group performance status assessment (ECOG); appetite loss; abnormal biochemistry (white blood cell count > 10 × 10^9^/L, albumin < 35 g/L, haemoglobin < 10 mg/dL] was used to classify cachexia (no cachexia, pre-cachexia, cachexia and refractory cachexia) (Zhou 2018).

### Sample collection, processing, and storage

Ten millilitres of blood were drawn per blood collection tube from each participant, where one ethylene diamine tetra acetic acid (EDTA)-containing tube was used for full blood count analysis, one serum separator tube (SST) was used for serum albumin determinations and one ethylene diamine tetra acetic acid (EDTA)-containing tube for the investigational markers (CRP, TNFα, IL-6, IL-8, CXCL5 and H3Cit). Blood samples for investigational markers (CRP, IL-6, IL-8, TNFα, H3Cit and CXCL5) were processed within 4 h of collection and the plasma fraction was aliquoted and stored at -80ºC (ThermoFisher Scientific Inc., Waltham, MA, USA) until use.

### Measurement of biomarkers

Biomarkers investigated included albumin, haemoglobin (Hb), white blood cell count (WBC) including circulating neutrophils, lymphocytes and platelets, CRP, IL-6, IL-8, TNFα, CXCL5, and H3Cit. Neutrophil to lymphocyte ratio (NLR), platelet to lymphocyte ratio (PLR) and the systemic immune inflammation index (SII) were calculated. Routine biomarkers (albumin, haemoglobin, neutrophil, lymphocyte and platelet counts) were analysed by Lancet Laboratories©, Johannesburg, South Africa, on the day of collection and standardized reference ranges were taken as the accepted ranges used by commercial pathology laboratories.

Tumour necrosis factor α, IL-6 and IL-8 concentrations were measured using a MILLIPLEX Map Cytokine/Chemokine kit (Merck KgaA, Darmstadt, Germany) and CXCL5 levels were determined using an Invitrogen ProcartaPlex Multiplex suspension-bead array assay (Thermo Fisher Scientific, Inc.). The assays were conducted according to the manufacturer’s instructions and a Bio-Plex suspension array reader (Bio-Rad Laboratories Inc., Hercules, CA, USA) together with Bio-Plex Manager software 6.0 was used for bead acquisition and analysis of median fluorescence intensity. The results are reported as picograms(pg)/millilitre(mL).

Levels of H3Cit were measured using the Clone11D3 enzyme-linked immunosorbent assay (ELISA) kit (Cayman Chemical Co., Ann Arbor, MI, USA) (Kit 2018). The method was followed as outlined by the manufacturer and the optical density for each sample was measured at 450 nm (nm) using a PowerWaveX spectrophotometer (BioTek Inc., Winooski, VT, USA). The results are presented as nanograms (ng)/mL.

C-reactive protein levels were determined using the CardioPhase hsCRP test kit (Siemens Healthcare Diagnostics, Midrand, Johannesburg, South Africa) and analysed using the Attelica 630N nephelometer (Siemens, MU, Germany). The CRP concentrations are reported as milligrams (mg)/Litre (L).

### Statistical analysis

Data Science Workbench, Version 14. MicroSoft Excel was used to capture the data, which was imported to STATISTICA 13, TIBCO Software Inc. (2020) for statistical analyses. Summary statistics and descriptive statistics were used to describe the variables. Medians or means were used as the measures of a central location for ordinal and continuous responses and quartiles and standard deviations as indicators of spread, respectively. Correlation between two continuous variables was measured with the Pearson correlation, or Spearman correlation. The relationship between discrete variables was investigated with contingency tables and chi-square tests.

Continuous variables were compared between the two groups using analysis of variance (ANOVA) or equivalently pooled *t* tests. If the variances of the two groups differed significantly, the Welch test was used. If the residuals from ANOVA were not normally distributed, the Mann–Whitney test was used as the non-parametric equivalent of the pooled *t* test.

For investigational markers and the measures of inflammation, where there are no formal accepted reference values, cut-offs yielded from receiver operating characteristic (ROC) curve analysis were applied to test for significance.

### Ethical clearance

Ethics approval was granted by the Stellenbosch University Health Research Ethics Committee (HREC) (Approval number S19/10/223). All participants gave written informed consent to participate in the study.

## Results

### Description of participants

**Fig. 1:**
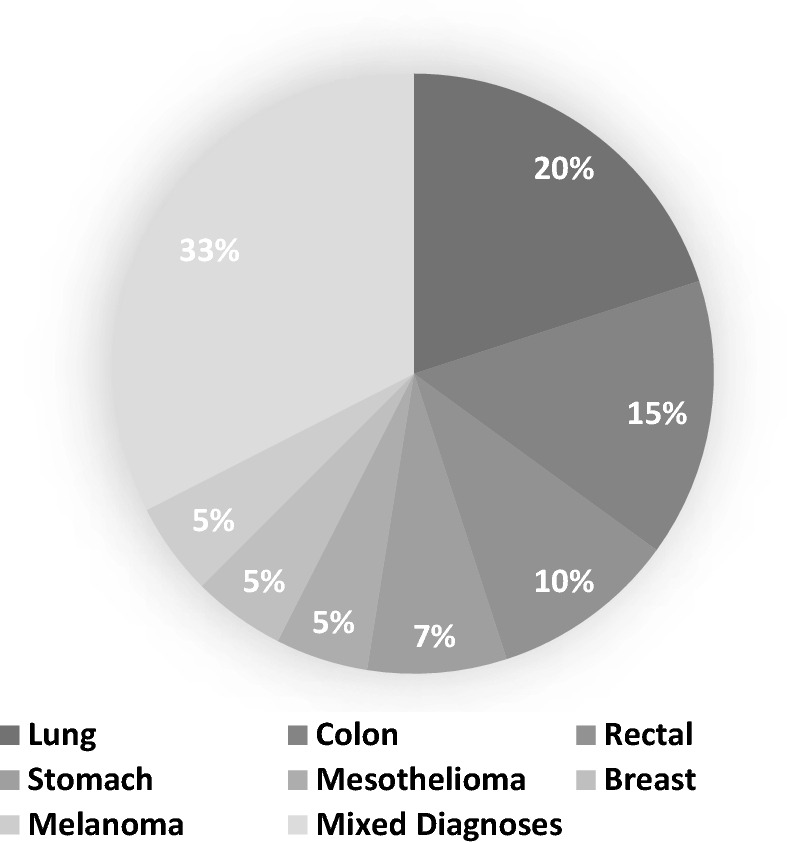
Schematic representation of the distribution of primary diagnoses of patients

No significant difference for age (*p* = 0.95) was found between the age- and gender-matched patient and control individuals. Approximately 70% of the participants were above the age of 60 years, with a mean age of 64.03 years (± 12.63), ranging from 30 to 88 years old. The participants were predominantly male (65% versus 35% females). The multiple diagnoses for patients included 16 different diagnoses as summarized in Fig. 1, with 33% of the patients presenting with various mixed diagnoses, 20% with lung cancer, 15% with colon cancer and 10% with rectal cancer as the primary diagnoses.

### Baseline anthropometrics

Anthropometric analyses yielded striking differences between patient and control individuals. The compromised nutritional status of the patients was evident in the significant differences for weight, BMI, protein, SMM, SMI and HGS, as shown in Table 1.

**Table 1:** Summary of measured anthropometric variables

Measurement	Patients	Controls	*p*-value
Weight (kg)			
Whole sample	68.46 (± 16.13)	81.18(± 13.12)	< 0.01
Males	74.70 (± 15.00)	85.37(± 12.82)	< 0.01
Females	56.87 (± 11.20)	73.40 (± 9.99)	< 0.01
Body Mass Index (kg/m^2^)			
Whole Sample	24.00 (± 4.50)	28.46 (± 3.74)	< 0.01
Males	25.16 (± 4.25)	28.59 (± 3.81)	< 0.01
Females	21.84 (± 4.25)	28.20 (± 3.72)	< 0.01
% Weight loss			
Patients total	16.15 (± 8.40)		
Male patients	14.50 (± 6.70)		
Female patients	19.21 (± 10.49)		
Protein (kg)			
Whole sample	9.43 (±2.26)	10.68 (±2.06)	0.01
Males	10.53 (±1.96)	11.73 (±1.65)	0.01
Females	7.37 (± 0.96)	8.75 (± 1.17)	0.03
Skeletal muscle mass (kg)			
Whole sample	26.45 (± 6.84)	30.27 (± 6.21)	0.01
Males	29.81 (± 5.93)	33.40 (± 4.97)	< 0.01
Females	20.22 (± 2.93)	24.45 (± 3.52)	0.02
Skeletal muscle index (kg/m^2^)			
Whole sample	6.67 (± 1.34)	7.67 (1.08)	< 0.01
Males	7.36 (± 1.06)	8.20 (± 0.81)	< 0.01
Females	5.38 (± 0.73)	6.68 (± 0.78)	< 0.01
Hand grip strength (kg)			
Whole sample	24.42 (± 9.53)	29.62 (± 8.45)	0.01
Males	27.38 (± 9.31)	33.98 (± 6.12)	< 0.01
Females	18.92 (± 7.47)	21.52 (± 5.79)	0.36

When analysing the BMI of the participants, 70% of the patients were classified as normal weight, overweight or obese. 73% (29 patients) reported weight loss greater than 10% in the past 6 months and were therefore classified as having *severe* weight loss, while 25% (10 patients) were classified as having *significant* weight loss (weight loss greater than 5%). Only 2.5% (1 patient reported no weight loss in the past 6 months (Gibson 2005).

According to defined cut-offs for sarcopenia, the male patients (SMI at 7.36(± 1.06) kg/m^2^) did not present with sarcopenia, notwithstanding that for males the SMI was significantly different for patients and control participants, (*p* < 0.01). Female patients, however, were defined as sarcopenic according to prescribed cut-offs with a mean SMI of  5.38 (± 0.73) kg/m^2^.

With respect to HGS, the patients showed significantly lower muscle strength (*p* = 0.01) than the control individuals. Analysing the genders separately, HGS scores for male patients were significantly lower compared to control participants (*p* < 0.01), while for females, HGS measurements did not differ significantly (*p* = 0.36). According to accepted cut-offs for the definition of sarcopenia, female patients were *not* classified as sarcopenic, however, male patients were defined as sarcopenic according to the cut-offs for HGS with the observed mean HGS for males being 27.38 (± 9.31) kg.

**Fig. 2:**
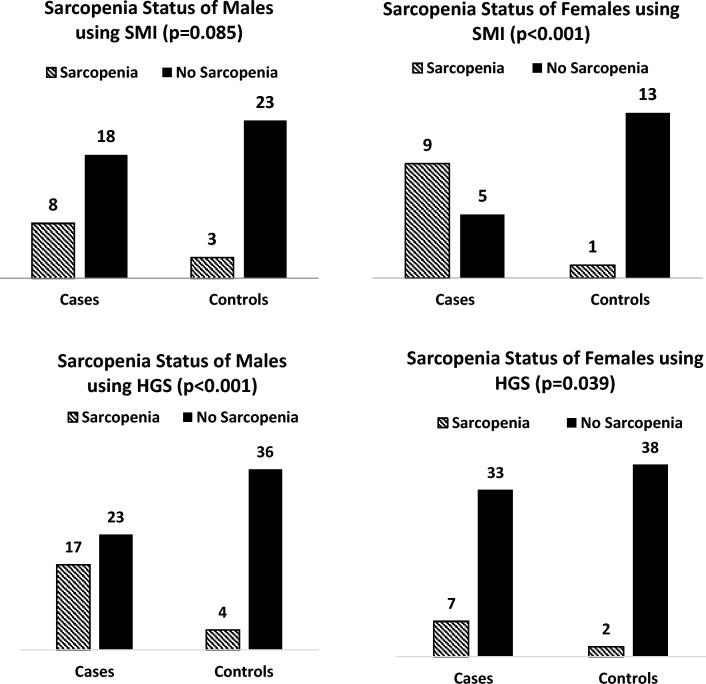
Presence of sarcopenia according to skeletal muscle index (SMI) and handgrip strength (HGS)

Sarcopenia, according to SMI and HGS, was further analysed in the context of the presence or absence of sarcopenia for each participant according to the given cut-offs. The patients had approximately four times the incidence of sarcopenia according to SMI [42.5% (*n* = 17) for patients versus 10% (*n* = 4) for controls], *p* < 0.001. For males, 30.8% (*n* = 8) of patients compared to 11.5% (*n* = 3) of controls presented with sarcopenia (*p* = 0.085) and for females, 64.2% (*n* = 9) of the patients compared to 7.1% (*n* = 1) of the control individuals were identified with sarcopenia (*p* < 0.001). Comparatively, the frequency of sarcopenia was approximately four times greater when assessing sarcopenia using HGS [60% (*n* = 24) versus the controls 15% (*n* = 6), *p* < 0.001]. For males, 65.4% (*n* = 17) of patients versus 15.4% (*n* = 4) of controls were defined as being sarcopenic, *p* < 0.001, while for females, 50% (*n* = 7) versus 14.3% (n = 2) of control participants were sarcopenic, *p* = 0.039. These results are depicted in Fig. 2.

**Fig. 3:**
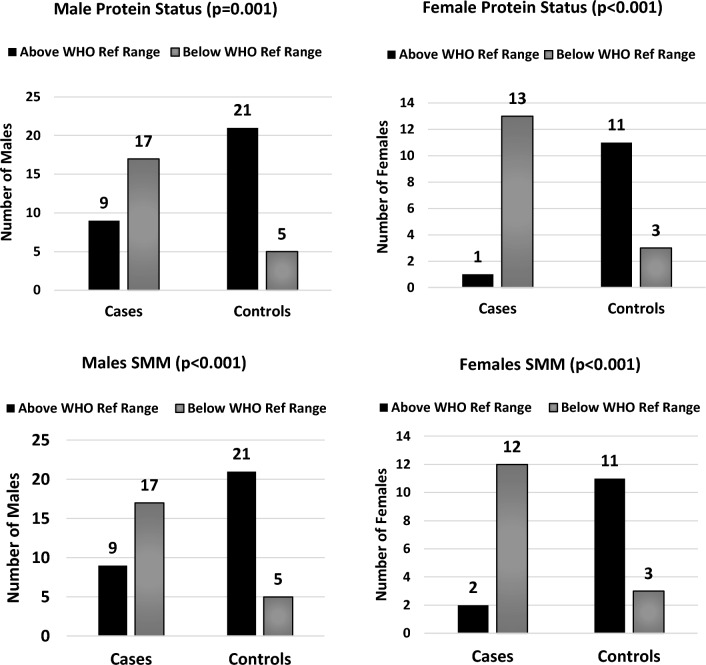
Protein and skeletal muscle mass (SMM) comparing cases to controls. *WHO* World Health Organization (“WHO: Global Database on Body Mass Index”)

With respect to total protein and SMM, significantly more female participants had values below the World Health Organisation’s (WHO) reference ranges (WHO Global Data Base), the levels of significance being *p* < 0.001 and *p* = 0.004, respectively. Ninety-three per cent (*n* = 13) of the patients, as opposed to 21% (*n* = 3) of the controls, were found to have a total protein below the WHO reference range, with SMM showing a similar trend, these results being 86% (*n* = 12) and 21% (*n* = 3) for patient and control individuals, respectively. Similarly, for males, significantly more patient than control participants were below the WHO reference ranges when assessing total protein levels (*n* = 17 for patients versus n = 5 for controls, *p* = 0.001) and SMM (*n* = 17 for patients versus *n* = 5 for controls, *p* < 0.001). The results of the protein and SMM assessments are represented in Fig. 3.

### Cachexia staging

**Fig. 4:**
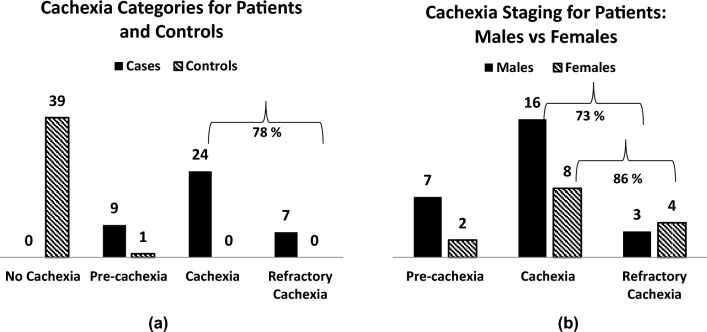
Number of patients and control individuals in the cachexia categories: Patients versus controls (**a**) and male versus female patients (**b**)

For cachexia scoring, mean CSS were significantly different for patient and control participants (*p* < 0.01). None of the control individuals were classified as cachectic. Grouping cachexia and refractory cachexia, 78% of the patients (*n* = 31), presented with both cachexia and refractory cachexia. Analysing genders separately, 73% of male patients and 86% of female patients were in the cachexia scoring classification categories of “cachexia” and “refractory cachexia”, according to the cachexia grading as defined by the cachexia scoring tool applied. Only 22.5% (*n* = 9) of the patients presented with pre-cachexia. The results of the cachexia assessments are depicted in Fig. 4.

### Biomarker assessment

All biomarkers without described and accepted cut-off values were assessed using ROC analysis and their respective area under curve (AUC) values were used for statistical analysis. Results of the ROC analysis are tabulated in Table 2.

**Table 2:** Summary of receiver operating characteristic (ROC) curves ranking

Rank	Continuous variable	AUC	Sensitivity (%)	Specificity (%)
1	PLR	0.84	80.00	87.50
2	CRP	0.80	80.00	80.00
3	TNFα	0.79	75.00	72.50
4	NLR	0.73	70.00	75.00
5	SII	0.72	62.50	82.50
6	IL-8	0.65	60.00	67.50
7	IL-6	0.64	82.50	55.00
8	CXCL5	0.59	67.50	57.50
9	H3Cit	0.56	65.00	55.00

*AUC* area under curve, *H3Cit* citrullinated histone H3, *CXCL5* C-X-C motif chemokine ligand 5, *CRP* C-reactive protein, *IL-6* Interleukin-6, *IL-8* Interleukin-8, *NLR* neutrophil to lymphocyte ratio, *PLR* platelet to lymphocyte ratio (*PLR*), *SII* Systemic Immune Inflammation Index, *TNFα* Tumour necrosis factor alpha

For the patients, the mean values for albumin, WBC, neutrophils, lymphocytes, and platelets were all within the given reference ranges, but were significantly different from the matched control participants; however, the mean Hb for the patients was below the reference value at 12.38 g per decilitre (g/dL). These results are summarized in Table 3.

**Table 3:** Blood marker analysis

Marker	Reference ranges	Patients mean (± SD)	Controls mean (± SD)	*p*-value for Significance (Patients versus Controls)	*p*-value for Significance (Patients versus Reference Constant)
Albumin (g/L)	35–50	39.66 (± 6.41)	46.99 (± 2.21)	*p* < 0.01	
WBC × 10^9^/L	4.0–12.0	7.89 (± 6.34)	7.07 (± 1.87)	*p* = 0.42	
Neutrophils × 10^9^/L	2.0–7.5	5.43 (± 5.60)	4.40 (± 1.65)	*p* = 0.27	
Lymphocytes × 10^9^/L	1.0–4.0	1.45 (± 0.73)	2.04 (± 0.60)	*p* < 0.01	
Platelets × 10^9^/L	150–450	300.34 (± 155.32)	231.00 (± 55.15)	*p* < 0.01	
Haemoglobin (g/dL)	13.8–18.8	12.38 (± 2.04)	15.13 (± 0.92)	*p* < 0.01	
NLR	2.73^a^	4.85 (± 6.59)	2.31 (± 1.10)	*p* = 0.02	*p* = 0.008
PLR	148.82^a^	232.90 (± 119.70)	119.18 (± 34.63)	*p* < 0.01	*p* < 0.001
SII	791.96^a^	1387.35 (1866.47)	543.54 (± 301.74)	*p* < 0.01	*p* = 0.051
CRP (mg/L)	2.775	31.65 (± 56.54)	2.78 (± 6.72)	*p* < 0.01	*p* = 0.002
TNFα (pg/mL)	20.745	43.52 (± 52.77)	15.69 (± 13.51)	*p* < 0.01	*p* = 0.009
IL-6 (pg/mL)	4.39	41.13 (± 6.87)	35.64 (± 69.07)	*p* = 0.04	*p* < 0.001
IL-8 (pg/mL)	9.175	33.08 (± 59.90)	29.85 (± 81.53)	*p* = 0.02	*p* = 0.023
CXCL5 (pg/mL)	42.28	91.37(± 140.30)	61.74 (± 59.01)	*p* = 0.22	*p* = 0.033
H3Cit (ng/mL)	1.295	2.38(± 2.88)	2.38 (± 6.72)	*p* = 0.99	*p* = 0.023

*H3Cit* citrullinated Histone H3, *CXCL5* C-X-C motif chemokine ligand 5, *IL-6* Interleukin-6, *IL-8* Interleukin-8, *NLR* neutrophil to lymphocyte ratio, *PLR* platelet to lymphocyte ratio, *SII* systemic immune inflammation index, *CRP* C-reactive protein, *TNFα* tumour necrosis factor alpha, *WBC* white blood cell count

^a^Threshold/cut-off from ROC curve

Markers of inflammation, including NLR, PLR and SII showed significant differences between patient and control individuals. Furthermore, in the case of the NLR and PLR values, these results were significantly greater than the reference cut-off’s with SII showing borderline significance to the reference values. These results are indicative of the metabolic signs and symptoms of advanced cachexia, in which routine markers of inflammation were elevated, while nutritional blood markers were low compared to the healthy, matched control participants. In the case of CRP, TNFα, IL-6, IL-8, CXCL5 and H3Cit, the results observed with the patients differed significantly from the ROC curve reference cut-offs. The results of the biomarker analyses are shown in Table 3.

### Relationships of biomarkers to nutritional status and cachexia

#### Sarcopenia and body composition

**Fig. 5:**
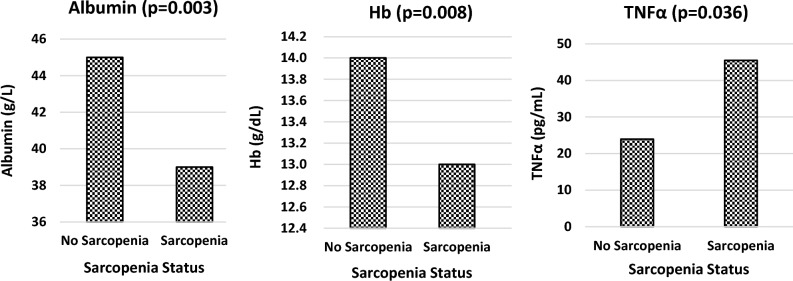
Relationships of albumin, haemoglobin (Hb) and tumour necrosis factor alpha (TNFα) to sarcopenia (*n* = 17) or no sarcopenia (*n* = 23) as defined by skeletal muscle index (SMI). Hb Haemoglobin, TNFα Tumour necrosis factor alpha

Correlation investigations revealed that the presence or absence of sarcopenia (defined by SMI cut-offs) was significantly related to albumin (*p* = 0.003), Hb (*p* = 0.008) and TNFα (*p* = 0.036) using continuous variables, depicted graphically in Fig. 5. Sarcopenia was also significantly correlated with weight (*p* < 0.001) and percentage weight loss (*p* = 0.034) for the patients.

Using cut-offs for biomarkers and categories for presence or absence of sarcopenia, the results showed that only CRP was significantly associated with the presence or absence of sarcopenia (*p* = 0.01). However, when the cut-off for sarcopenia was analysed by ANOVA in relation to the continuous variables of the biomarkers, the results showed that sarcopenia correlated significantly with albumin (*p* < 0.01), Hb (*p* < 0.01), TNFα (*p* = 0.02) and CRP (*p* < 0.01).

Total body protein was significantly correlated with Hb (*p* < 0.001) and albumin (*p* = 0.002). Both SMI and SMM were significantly related with WBC, albumin and Hb, but no significant relationships were found with any of the other routine markers, markers of inflammation or investigational markers. With respect to total protein and SMM in relation to the continuous variables, significance was found with albumin, Hb and TNFα for both parameters, with significance determined at *p* < 0.01 for all three.

Analysis of biomarkers categorized as being either “above” or “below” the cut-offs as per the ROC curves and total body protein and SMM as either “above” or “below” the WHO midpoint reference point, NLR (*p* = 0.04), PLR (*p* = 0.01), TNFα (*p* = 0.01) and H3Cit (*p* = 0.04) were found to show significant associations with total protein, while for SMM, significant associations with PLR (*p* = 0.02) and TNFα (*p* = 0.02) were found.

#### Hand grip strength

**Fig. 6:**
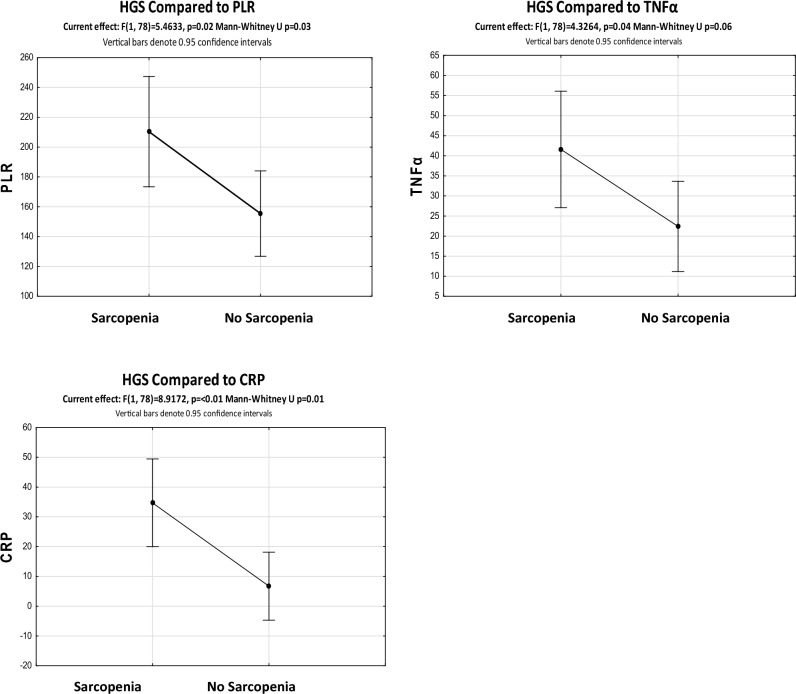
Significant relationships of platelet to lymphocyte ratio (PLR), tumour necrosis factor alpha (TNFα), and C-reactive protein (CRP) to the presence or absence of sarcopenia as defined by hand grip strength (HGS) (*n* = 80). *CRP* C-reactive protein, *HGS* hand grip strength, *PLR* platelet to lymphocyte ratio, *TNFα* tumour necrosis factor alpha

Both albumin (*p* < 0.01, *r* = 0.45) and Hb (*p* < 0.001, *r* = 0.44) correlated significantly with HGS, but no significant relationships were demonstrated between HGS and any of the other biomarkers investigated. For nominal comparisons of HGS to cut-offs of biomarkers, no significant differences were found for HGS with any of the biomarkers. However, when HGS categories were compared to continuous variables for biomarker described cut-offs, significances were observed with respect to PLR, TNFα, and CRP. These results are presented in Fig. 6.

#### Cachexia status

**Fig. 7:**
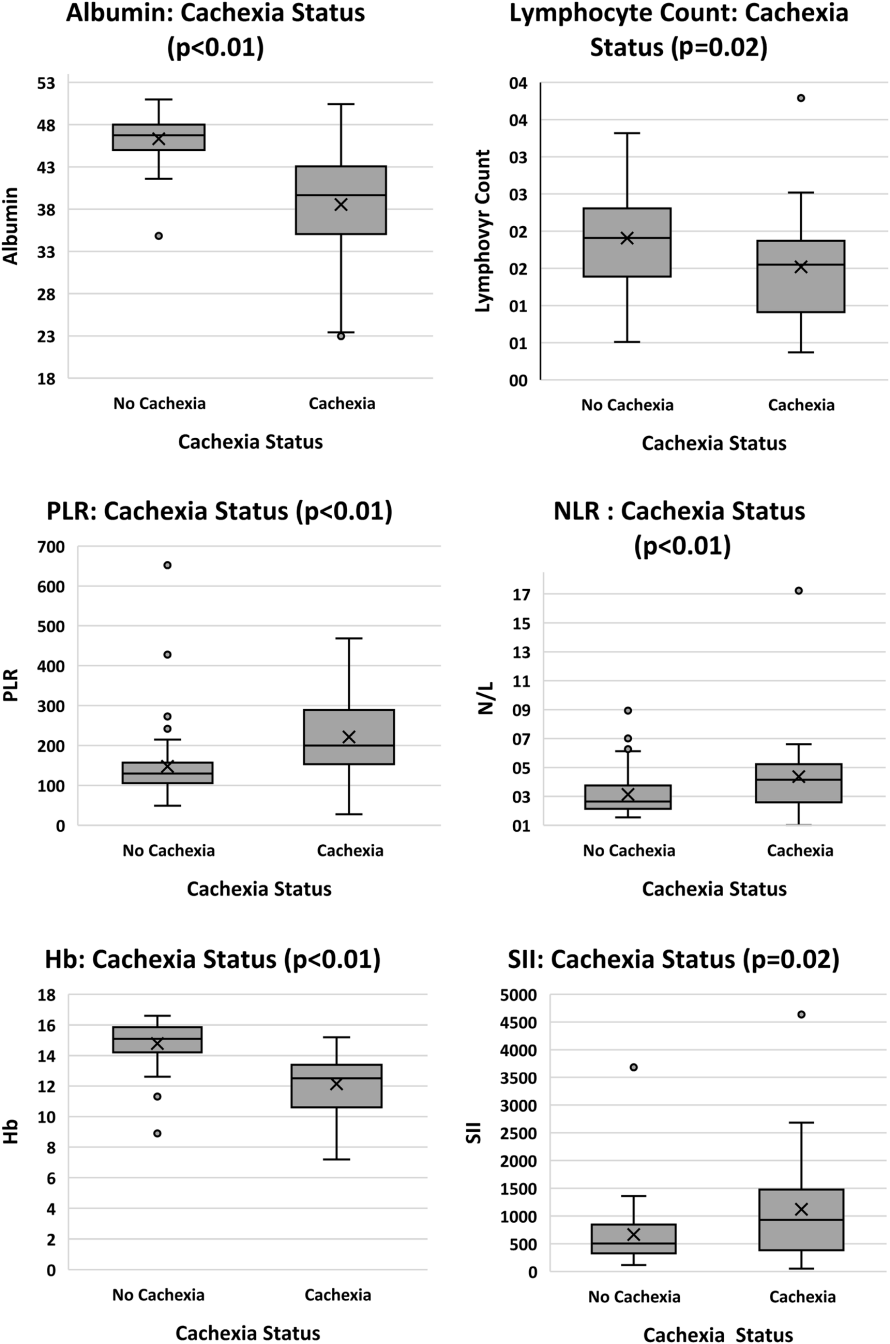

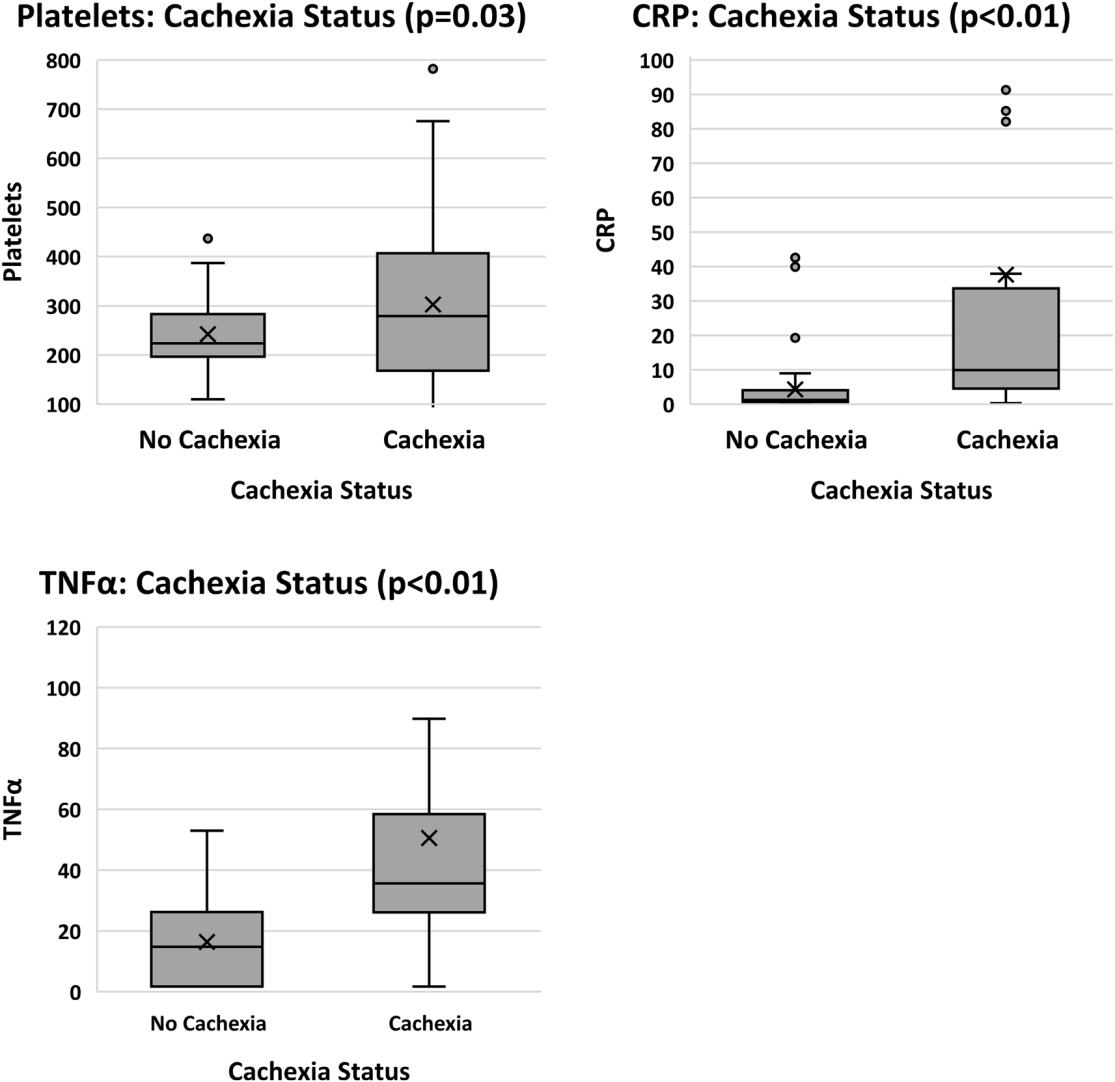
Biomarkers in relation to cachexia status (*n* = 80) where participants were categorized as either having cachexia present or absent. *CRP* C-reactive protein, *Hb* haemoglobin, *IL-6* interleukin-6, *NLR* neutrophil to lymphocyte ratio, *PLR* platelet to lymphocyte ratio, *TNFα* tumour necrosis factor alpha

Regarding CSS scores, positive significant relationships were found for NLR, PLR, SII, TNFα, IL-6, IL-8 and CRP. Significant negative relationships were shown for albumin, Hb and lymphocyte counts. No significance was observed with neutrophils, platelets, WBC, CXCL5 or H3Cit. When relationships were investigated for cachexia categories, CSS scores were grouped into two sub-groups: “cach” (cachexia present) and “none” (cachexia absent). Cachexia and refractory cachexia were grouped as “with” cachexia, and no-cachexia  and pre-cachexia were grouped as “without”. Analysis was completed using two approaches. Firstly, CSS categories were investigated according to the continuous values for biomarkers (where significance was found for albumin, lymphocytes, NLR, Hb, platelets, PLR, SII, TNFα, and CRP) as shown in Fig. 7. Secondly, categories of CSS were compared with biomarkers using nominal variables with respect to the reference constants for each biomarker. Significant differences between CSS categories were found for all investigational markers except CXCL5 and H3Cit.

## Discussion

Cancer cachexia alone may account for more than 20% of deaths in patients. Cachexia is present in 15 to 40% of cancer patients affecting approximately 80% of patients with advanced illness (Mondello et al. 2014). In the current study, 60% of the patients presented with cachexia, 17.5% with refractory cachexia and 9.5% with pre-cachexia results that are consistent with those described in the literature and therefore, the metabolic derangements associated with cancer cachexia were expected to be present.

Ideal cachexia scoring should include facets of inflammation, weight loss, sarcopenia and appetite for optimal classification (Zhou et al. 2018), and not percentage weight loss and BMI exclusively (Crawford 2019). Cachexia staging identifies periods of stability, where the potential for reversal of muscle loss (even 90 days preceding death) (Prado et al. 2013) is possible and detects patients who may respond to nutritional intervention during this “window”, enabling nutritional and medical goal-directed treatment, that is, interventions affecting prognosis versus palliative care (Ozorio et al. 2017).

Sarcopenia, measured by bioelectrical impedance analysis (BIA) identifies patients with, or at risk of, developing cachexia (Crawford 2019). The presence of sarcopenia observed in this group of patients (42% for SMI versus 60% for HGS) would place them at greater risk for cachexia. BMI showed that 35% of the patients were overweight and 10% were obese, therefore, masking the underlying sarcopenia. Sarcopenia, independently, is prognostic for lower survival in obese patients with cancer (Martin et al. 2015), therefore, its assessment is crucial for a thorough nutritional assessment.

Haemoglobin (Hb) and serum albumin have been extensively investigated as biomarkers of malnutrition in cancer patients (Martin et al. 2015; Bullock et al. 2020; Knight et al. 2004). Decreased serum albumin reflects lean tissue loss, an increased systemic inflammatory response and may be a negative prognostic factor for survival in various primary cancer diagnoses (Bullock et al. 2020). The significantly lower albumin value of the patients versus that of the controls’, is suggestive of the advanced nature of the cachexia in this study. Additionally, the significantly higher presence of anaemia where, 78% of the patients were anaemic, is similar to that described in the literature (30–90% of patients are anaemic). The significantly lower Hb in this study renders Hb a reliable biomarker, indicative of the extent of inflammation and cachexia, that may be caused by a plethora of factors (Madeddu et al. 2018).

Neutrophils, lymphocytes and platelets play a role in systemic inflammation, where their respective ratios, provide valuable insights regarding prognosis, staging and metastasis (Yang et al. 2018). Elevated blood neutrophil counts and reduced lymphocyte counts are typical in inflammation (Dupré and Malik 2018). The results in the current study did not reflect this: neutrophil levels were not raised in the cases; however, lymphocyte counts were significantly lower, in keeping with the presence of inflammation. Numerous factors may affect neutrophil counts, including blood volume, ethnicity, age, extent of disease, treatment status with administration of chemotherapy drugs and smoking status, some of which were not controlled for in this study (Wu et al. 2020).

Platelets may promote tumour growth and metastasis (Gianazza et al. 2020) and may also predict survival in cancer cachexia. Wang et al. showed that a platelet count > 261 × 10^9^/L was associated with a reduced overall survival (Wang et al. 2020). Comparatively, the mean platelet count of the patients in this study was 300 × 10^9^/L, indicating advanced cachexia and disease, supporting emerging data on the diagnostic potential of platelet counts in cancer cachexia (Haemmerle et al. 2018).

Increased NLR and PLR are correlated with a decreased overall survival and cancer-specific survival (Dupré and Malik 2018) and a raised SII may indicate a poor prognosis in various malignancies (Hu et al. 2014), being potentially more reliable than NLR and PLR (Hirahara et al. 2020). NLR, PLR and SII were all significantly higher for the patients compared to the controls in this study further supporting the evidence of the advanced nature of the disease.

For the patients, investigational markers (CRP, IL-6, IL-8 and TNFα) all were significantly higher in relation to the reference values obtained for ROC curve analysis. These results underpin the protein depletion evident in the patients, with these biomarkers driving the nutritional deterioration. Tumour necrosis factor alpha plays a role in muscle protein breakdown, raised IL-6 affects muscle mass (Carson and Baltgalvis 2010), and an overexpression of IL-8 is correlated with metastases and advanced disease (Alfaro et al. 2017)—synonymous with a reduced nutritional status. Associations of these cytokines to sarcopenia is central to cachexia management in the early stages, guiding clinicians to direct treatment plans as aggressive or palliative (Paczek et al. 2020), dependent on how advanced the disease is as reflected by the biomarkers.

Research that documents raised CXCL5 levels may be questionable due to inconsistencies in its measurement (Binwu 2018), challenging the reliability of CXCL5 as a prognostic marker for cancer (Hu et al. 2018). Plausible explanations for differences found in the reporting levels of CXCL5 levels include: measurement of tissue samples versus serum levels; biomarker measurement in peripheral circulation versus blood surrounding the tumour samples; tumour stage [CXCL5 levels are dependent on stages and not tumour size or mass (Lim and Chung 2015)]; the extent of metastasis when measurement occurred (Hu et al. 2018); small sample sizes (Lee et al. 2018). These areas of inconsistency are not standardized across all literature, questioning the prognostic value of CXCL5 expression in cancer patients. These confounding variables emphasize the need for larger and more clearly standardized research approaches for the investigations of CXCL5. The aforementioned factors may account for the absence of increased CXCL5 found in the patients compared to the control individuals and “poor performance” of CXCL5 in the ROC curve analysis (AUC 0.59) encountered in the current study.

Additionally, the timing and trajectory of the metastatic process in which the blood samples were collected may be relevant. Reports show that when CXCL5 and IL-8 are concurrently depleted, there is a synergistic effect on metastasis, where metastatic activity is increased (Lopez-Lago et al. 2013). Therefore, the rise and fall of CXCL5 and IL-8 may impact results depending on the respective trajectories of the metastatic process. The flux and interplay between biomarkers is not completely understood.

The significantly raised IL-8 and “normal” CXCL5 levels found in the patients in this study could be an indication that the environment was not conducive for enhanced metastatic activity. Potentially, results may have been different if the samples were taken a few weeks earlier or later. This raises the question of the timing in measuring these biomarkers, i.e., in isolation or as consecutive samples at defined time intervals. Biomarker levels could possibly rise and fall in “waves” and “cycles” dependently or independently to drive metastasis, impacting the reliability of these cytokines as cachexia indicators and furthermore, making objective comparisons of the relationships of these markers to sarcopenia between studies challenging.

The results of H3Cit in this study also differed from those reported in the literature. No notable difference was found between patients and control participants, and the AUC for ROC curve analysis was 0.56. In contrast, others have reported raised (three-fold) H3Cit levels in cancer patients (Thålin et al. 2018). This difference may be explained by several factors: choice of methodology used for analysis (serum versus colorectal mucus samples) and single diagnosis as opposed to mixed diagnoses (Loktionov et al. 2019). Furthermore, other studies reported that approximately 20% of cancer patients displayed neutrophil counts of more than double the upper reference limit (Thålin et al. 2018), which was not the case in the current study, possibly explained by differences in stages of chemotherapy of the patients. Neutrophil activation and neutrophil extracellular trap (NET) formation is a probable source of circulating H3Cit in cancer patients (Thålin et al. 2018), perhaps the “normal” levels of circulating H3Cit found in this study are related to the “normal” neutrophil counts shown.

Lack of standardization in cut-offs and reference values further complicates comparisons. Some studies use a cut-off at the 75th percentile (Grilz et al. 2019), but omit ROC curve analysis, while others report high AUC results (0.884) for H3Cit, where H3Cit was investigated as part of the peptidylarginine deiminase 4 (PAD4) complex, inclusive of other markers (Loktionov et al. 2019). Additionally, currently used ELISAs are not standardised for H3Cit measurement (Thålin et al. 2020). These inconsistencies in the measurement and reporting may explain why the outcomes in this study differ from those reported in the literature.

Patient selection may also impact H3Cit outcomes. Studies reporting raised H3Cit focussed primarily on patients in the early stages of their disease (Grilz et al. 2019; Loktionov et al. 2019), therefore, questioning the extent of metastatic disease and presence of cachexia (Thålin et al. 2018). The presence of cachexia in 60% of the patients in the current study may account for the differences shown in this study. Furthermore, H3Cit levels are elevated in diagnoses including strokes, COVID-19 and aortic stenosis. Studies reporting raised H3Cit did not exclude these conditions (Thålin 2018), which too may confound results. Therefore, specific guidelines for future research regarding exclusions for measurement of H3Cit, requires clarification.

“Resistance” and “tolerance” are terms used to describe the underlying physiology and metabolic changes of cancer cachexia stages (Maccio et al. 2021). The resistance phase is an initiation of the immune response to target tumour or cancer cells, accompanied by inflammation. The tolerance phase aims to mitigate the damage caused by the resistance phase. The progression from resistance to tolerance and flux between these phases is not clearly defined, driven by factors that are not fully understood and may impact on the measurements of biomarkers. Therefore, markers that indicate the extent of resistance versus tolerance phases are required before attempting to understand the role of biomarkers that drive metastasis and cachexia. These considerations remain largely unknown complicating standardisation for future research.

Both albumin and Hb showed significant relationships to HGS and protein status, where lower biomarkers levels related significantly to lower HGS, total protein, SMM, and SMI. Additionally, albumin was significantly associated with the presence or absence of sarcopenia. Haemoglobin was shown to be reliable in predicting malnutrition, and conversely sarcopenia was indicative of the presence of anaemia. Similarly, Wang et al. reported significantly lower albumin levels (*p* < 0.001) in the presence of sarcopenia in patients with advanced cancer (Wang et al. 2020).

Neither NLR, PLR nor SII were found to show significant associations with sarcopenia in the current study using SMI. Similarly, Laing et al. failed to show a significant relationship between NLR (*p* = 0.630) or PLR (*p* = 0.529) to sarcopenia, but did show significance for the association of the lymphocyte to monocyte ratio (LMR), *p* = 0.007 with sarcopenia (Liang et al. 2021). However, other studies have shown NLR (*p* = 0.011), lymphocyte count (*p* = 0.002) (Kim et al. 2016), and PLR (*p* < 0.001) (Chen 2018) (Lin et al. 2018) to be significantly associated with sarcopenia. In the current study, using ROC curve cut-offs to categorize participants for nominal statistical analysis, PLR was significantly correlated with total protein and SMM. No significance was found for NLR and SII using these parameters.

Using nominal category cut-offs, HGS was significantly associated with PLR, but no significance was shown for NLR or SII. Chen et al. showed no significance for HGS with NLR or PLR in their study, even though the same method for the cut-offs for HGS were applied, potentially due to the low AUC score for these biomarkers that was evident in their trial (Chen et al. 2020). Linking inflammation to cachexia status remains inconsistent, possibly attributed to the multiple definitions used to define cancer cachexia or the cuts-offs applied for statistical analysis.

Cut-offs of 10 mg/L for CRP in cancer cachexia typically yield significant relationships of CRP with a reduced lean mass and an increased loss of lean mass (Cordeiro et al. 2020). The current study found no significant associations of CRP with total protein, SMM or SMI; however, CRP was significantly related to sarcopenia and to HGS using the cut-off from the ROC curve analysis for CRP (2.775 mg/l). Perhaps this more “stringent” cut-off that was used from the ROC curve analysis, may explain why no significance was shown for CRP to total protein, SMM or SMI in this study.

For IL-6, and TNFα, the literature supports relationships of these markers with anorexia and skeletal muscle breakdown (Kim et al. 2016). While significantly raised IL-6 (*p* < 0.0001), TNFα (*p* < 0.0001), and CRP (*p* < 0.0001) in malnourished and at-risk for malnutrition patients with colorectal cancer using the mini-nutrition assessment score (MNA) has been reported (Daniele et al. 2017), in gastric and lung cancer patients, IL-6 failed to predict weight loss and sarcopenia, even as its concentration increased. The authors suggested that IL-6 may be more valuable in the early stages of cancer cachexia with increased acute phase proteins causing tissue wasting (Scheede-Bergdahl et al. 2012). In the current study, significant associations of IL-6 to malnutrition were only found using ROC curve cut-offs for the biomarker and categories for the malnutrition assessment. Similarly, applying cut-offs for total protein and SMM, TNFα was the only biomarker found to be significantly associated with protein status, sarcopenia and with the presence or absence of sarcopenia. Furthermore, significant relationships were observed for both IL-6 and TNFα with HGS. In contrast, other research yielded no meaningful relationships between anthropometric measurements and IL-6 when using continuous variables for statistical analyses (Srdic et al. 2016). These differences indicate that stage of disease or statistical methods applied may be factors affecting outcomes and interpretation of results.

Cachexia and sarcopenia definitions may impact outcomes in correlating these measurements to IL-8. Whilst, higher levels of IL-8 have positively correlated with weight loss and sarcopenia in pancreatic cancer [percentage weight loss to define cachexia (> 5%) and computed tomography (CT) images for the diagnosis of sarcopenia] (Hou et al. 2018), the current study found that IL-8 was only significantly related with CSS categories, but not with sarcopenia, HGS, or protein status, potentially explained by differences in cachexia and sarcopenia definitions employed.

The literature supports significantly raised CRP (*p* = 0.020) and IL-6 (*p* = 0.040) for patients with cachexia compared to non-cachectic patients (Srdic et al. 2016). Similarly, using CSS scores, the current study found cachexia to be significantly related to CRP, IL-6, IL-8 and TNFα. All relationships were positive, indicating that as inflammation increased, the cachexia status deteriorated. Notwithstanding the poor ROC curve outcomes, H3Cit was significantly related to total protein when cut-offs for protein were applied, showing promise for future investigations for the use of H3Cit in cancer cachexia.

Relationships between CXCL5 and H3Cit to sarcopenia, anthropometric indices and cachexia are unexplored, with this study pioneering an attempt to find relationships in this regard. In this context, CXCL5 and H3Cit are considered relatively new and less well-described biomarkers for cancer cachexia. Inconsistencies in their respective measurements, and how they are integrated into research, requires standardization to enable the potential roles of these emerging biomarkers to be better interpreted from future research.

Although CXCL5 and H3Cit were not found to be reliable markers in cancer cachexia or with respect to their relationships to sarcopenia, this study introduces the paradigm of improving current knowledge of the relationships between the less understood biomarkers of advanced cancer cachexia. For future research, more clearly defined patient groups, with regard to stage of disease, primary diagnosis, presence of metastases, and including a comprehensive cachexia assessment and staging will produce more meaningful comparisons amongst studies and ensure goal-directed treatment for prognosis in cancer cachexia. The current study had limiting components including multiple different primary diagnoses of cases, the relatively small sample size to allow for powerful statistical analyses of smaller groups within the total sample and the diverse cancer treatment that cases were receiving. However, the value of this type of research coveys a message to the broader cachexia research community, to continue investigations to better understand emerging biomarkers, their measurement, together with sarcopenia and cachexia in more uniformly standardized settings.

## Data Availability

The datasets generated during and/or analysed during the current study are available from the corresponding author on reasonable request.

## References

[CR1] Alfaro C, Sanmamed M, Rodríguez-Ruiz M, Teijeira A, Oñate C, González A, Ponz M, Schalper K, L. Pérez-Gracia LJ, Melero I (2017) Interleukin-8 in cancer pathogenesis, treatment and follow-up. Cancer Treat Rev 60:24–31. 10.1016/j.ctrv.2017.08.004 28866366 10.1016/j.ctrv.2017.08.004

[CR2] Aoyagi T, Terracina K, Raza A, Matsubara H, Takabe K (2015) Cancer cachexia, mechanism and treatment. World J Gastroint Oncol 7(4):17–29. 10.4251/wjgo.v7.i4.17 10.4251/wjgo.v7.i4.17 PMC4398892 25897346

[CR3] Argilés J, Busquets S, Javier L (2019) Cancer cachexia, a clinical challenge. Curr Opin Oncol 31(4):286–290. 10.1097/CCO.0000000000000517 30893144 10.1097/CCO.0000000000000517

[CR4] Brocco D, Di Marino P, Grassandonia A (2019) From cachexia to obesity: the role of host metabolism in cancer immunotherapy. Curr Opin Support Palliat Care 13(4):305–310. 10.1097/SPC.0000000000000457 31389842 10.1097/SPC.0000000000000457

[CR5] Bruggeman AR, Kamal AH, LeBlanc TW, Ma JD, Baracos VE, Roeland EJ (2016) Cancer cachexia: beyond weight loss. J Oncol Pract 12(11):1163–1171. 10.1200/JOP.2016.016832 27858548 10.1200/JOP.2016.016832

[CR6] Bullock A, Greenley S, McKenzie G, Paton L, Johnson M (2020) Relationship between markers of malnutrition and clinical outcomes in older adults with cancer: systematic review, narrative synthesis and meta-analysis. Eur J Clin Nutr 74(11):1519–1535. 10.1038/s41430-020-0629-0 32366995 10.1038/s41430-020-0629-0 PMC7606134

[CR7] Carson J, Baltgalvis K (2010) Interleukin 6 as a key regulator of muscle mass during cachexia. Exerc Sport Sci Rev 38(4):168–176. 10.1097/JES.0b013e3181f44f11 20871233 10.1097/JES.0b013e3181f44f11 PMC3065300

[CR8] Chen L, Woo J, Assantachai P et al (2020) Asian working group for sarcopenia: 2019 consensus update on sarcopenia diagnosis and treatment. J Am Med Di Assoc 21(3):300–307. 10.1016/j.jamda.2019.12.012 10.1016/j.jamda.2019.12.012 32033882

[CR9] Cordeiro L, Silva T, Costa de Oliveira L, Nogueira Neto J (2020) Systemic inflammation and nutritional status in patients on palliative cancer care: A systematic review of observational studies. Am J Hosp Palliat Med 37(7):565–571. 10.1177/1049909119886833 10.1177/1049909119886833 31736322

[CR10] Crawford J (2019) What are the citeria for response to cachexia treatment ? Ann Palliat Med 8(1):43–49. 10.21037/apm.2018.12.08 30685983 10.21037/apm.2018.12.08

[CR11] Daniele A, Divella R, Abbate I, Casamassima A, Garrisi V, Savino E, Casamassima P, Ruggieri E, De Luca R (2017) Assessment of nutritional and inflammatory status to determine the prevalence of malnutrition in patients undergoing surgery for colorectal carcinoma. Anticancer Res 37(3):1281–1287. 10.21873/anticanres.11445 28314293 10.21873/anticanres.11445

[CR12] Dev R (2018) Measuring cachexia—diagnostic criteria. Ann Palliat Med. 8(1):24–32. 10.21037/apm.2018.08.07 30525765 10.21037/apm.2018.08.07

[CR13] Dupré A, Malik H (2018) Inflammation and cancer: What a surgical oncologist should know. Eur J Surg Oncol 44(5):566–570. 10.1016/j.ejso.2018.02.209 29530345 10.1016/j.ejso.2018.02.209

[CR14] Fearon K, Glass D, Guttridge D (2012) Review cancer cachexia: mediators, signaling, and metabolic pathways”. Cel Metab 16(2):153–166. 10.1016/j.cmet.2012.06.011 10.1016/j.cmet.2012.06.011 22795476

[CR15] Fearon K, Arends J, Baracos V (2013) Understanding the mechanisms and treatment options in cancer cachexia. Nat Rev Clin Oncol 10(2):90–99. 10.1038/nrclinonc.2012.209 23207794 10.1038/nrclinonc.2012.209

[CR16] Fiala O, Pesek M, Finek J, Racek J, Minarik M, Benesova L, Bortlicek Z, Sorejs O, Kucera R, Topolcan O (2016) Serum albumin is a strong predictor of survival in patients with advanced-tage non-small cell lung cancer treated with erlotinib. Neoplasma 63(3):471–476. 10.4149/318_151001N512 26952513 10.4149/318_151001N512

[CR17] Gianazza E, Brioschi M, Baetta R, Mallia A, Banfi C, Tremoli E (2020) Platelets in healthy and disease states: from biomarkers discovery to drug targets identification by proteomics. Int J Mol Sci 21(12):1–44. 10.3390/ijms21124541 10.3390/ijms21124541 PMC7352998 32630608

[CR18] Gibson R (2005) Principles of nutritional assessment. Oxford University Press, New York

[CR19] Grilz E, Mauracher L, Posch F, Königsbrügge O, Zöchbauer-Müller S, Marosi C, Lang I, Pabinger I, Ay C (2019) Citrullinated histone H3, a biomarker for neutrophil extracellular trap formation, predicts the risk of mortality in patients with cancer. Br J Haematol 186(2):311–320. 10.1111/bjh.15906 30968400 10.1111/bjh.15906 PMC6618331

[CR20] Haemmerle M, Stone R, Menter D, Afshar-Kharghan V, Sood A (2018) The platelet lifeline to cancer: challenges and opportunities. Cancer Cell 33(6):965–983. 10.1016/j.ccell.2018.03.002 29657130 10.1016/j.ccell.2018.03.002 PMC5997503

[CR21] Hirahara N, Matsubara T, Fujii Y, Kaji S, Kawabata Y, Hyakudomi R (2020) Comparison of the prognostic value of immunoinflammation-based biomarkers in patients with gastric cancer. Oncotarget 11(27):2625–2635 32676164 10.18632/oncotarget.27653 PMC7343633

[CR22] Hou Y, Wang C, Chao J, Chen Y, Wang H, Tung H, Lin J, Shan Y (2018) Elevated serum interleukin-8 level correlates with cancer-related cachexia and sarcopenia: an indicator for pancreatic cancer outcomes. J Clin Med 7(12):502–519. 10.3390/jcm7120502 30513776 10.3390/jcm7120502 PMC6306800

[CR23] Hu B, Yang X, Xu Y, Sun Y, Sun C, Guo W, Zhang X, Wang W, Qiu S, Zhou J, Fan J (2014) Systemic immune-inflammation index predicts prognosis of patients after curative resection for hepatocellular carcinoma. Clin Cncer Res 20(12):6212–6223. 10.1158/1078-0432.CCR-14-0442 10.1158/1078-0432.CCR-14-0442 25271081

[CR24] Hu B, Fan H, Lv X, Chen S, Shao Z (2018) Prognostic significance of CXCL5 expression in cancer patients: a meta-analysis. Cancer Cell Int. 10.1186/s12935-018-0562-7 29743818 10.1186/s12935-018-0562-7 PMC5930840

[CR25] Jones T, Stephenson K, King J, Knight K, Marshall T, Scott W (2009) Sarcopenia—mechanisms and treatments. J Geriatr Phys Ther 32(2):39–45 20039588

[CR26] Kim E, Kim YS, Seo JY, Park I, Ahn HK, Kim JH, Kim N (2016) The relationship between sarcopenia and systemic inflammatory response for cancer cachexia in small cell lung cancer. PLoS ONE 11(8):1–10. 10.1371/journal.pone.0161125 10.1371/journal.pone.0161125 PMC4990336 27537502

[CR27] Kit, Clone Elisa (2018) Citrullinated Histone H3 (Clone 11D3) ELISA Kit. 3 (501620)

[CR28] Knight K, Wade S, Balducci L (2004) Prevalence and outcomes of anemia in cancer: a systematic review of the literature. Am J Med 116(7 SUPPL. 1):11–26. 10.1016/j.amjmed.2003.12.008 10.1016/j.amjmed.2003.12.008 15050883

[CR29] Lee SJ, Kim JE, Kim ST, Lee J, Park SH, Park JO, Kang WK, Park YS, Lim HY (2018) The correlation between serum chemokines and clinical outcome in patients with advanced biliary tract Cancer. Transl Oncol 11(2):353–357. 10.1016/j.tranon.2018.01.007 29448202 10.1016/j.tranon.2018.01.007 PMC5852407

[CR30] Lerner L, Hayes TG, Tao N, Krieger B, Feng B, Wu Z, Nicoletti R, Chiu MI, Gyuris J, Garcia JN (2015) Plasma growth differentiation factor 15 is associated with weight loss and mortality in cancer patients. J Cachexia Sarcopenia Muscle 15(April):317–324. 10.1002/jcsm.12033 10.1002/jcsm.12033 PMC4670740 26672741

[CR31] Liang H, Peng H, Chen L (2021) Prognostic value of sarcopenia and systemic inflammation markers in patients undergoing definitive radiotherapy for esophageal cancer. Cancer Manag Res 13:181–192. 10.2147/CMAR.S288522 33469362 10.2147/CMAR.S288522 PMC7810973

[CR32] Lim JB, Chung HW (2015) Serum ENA78/CXCL5, SDF-1/CXCL12, and their combinations as potential biomarkers for prediction of the presence and distant metastasis of primary gastric cancer. Cytokine 73(1):16–22. 10.1016/j.cyto.2015.01.010 25689618 10.1016/j.cyto.2015.01.010

[CR33] Lin J, Zhang W, Huang Y, Chen W, Wu R, Chen X, Lou N, Wang P (2018) Sarcopenia is associated with the neutrophil/lymphocyte and platelet / lymphocyte ratios in operable gastric cancer patients: a prospective study. Cancer Manag Res 10:4935–4944. 10.2147/CMAR.S175421 30464594 10.2147/CMAR.S175421 PMC6208548

[CR34] Loktionov A, Soubieres A, Bandaletova T, Mathur J, Poullis A (2019) Colorectal cancer detection by biomarker quantification in noninvasively collected colorectal mucus: Preliminary comparison of 24 protein biomarkers. Eur J Gastroenterol Hepatol 31(10):1220–1227. 10.1097/MEG.0000000000001535 31498281 10.1097/MEG.0000000000001535

[CR35] Lopez-Lago MA, Posner S, Thodima VJ, Molina AM, Motzer RJ, Changanti RSK (2013) Neutrophil chemokines secreted by tumor cells mount a lung antimetastatic response during renal cell carcinoma progression”. Oncogene 32(14):1752–1760. 10.1038/onc.2012.201.Neutrophil 22665059 10.1038/onc.2012.201 PMC3435490

[CR36] Loumaye A, Thissen JP (2017) Biomarkers of cancer cachexia. Clin Biochem 50(18):1281–1288. 10.1016/j.clinbiochem.2017.07.011 28739222 10.1016/j.clinbiochem.2017.07.011

[CR37] Maccio A, Sanna E, Neri M, Oppi S, Madeddu C (2021) Cachexia as evidence of the mechanisms of resistance and tolerance during the evolution of cancer disease”. Int J Mol Sci 22:2890. 10.3390/ijms22062890 33809200 10.3390/ijms22062890 PMC8001015

[CR38] Madeddu C, Gramignano G, Astara G, Demontis R, Sanna E, Atzeni V, Macciò A (2018) Pathogenesis and treatment options of cancer related anemia: perspective for a targeted mechanism-based approach”. Front Physiol 9(SEP):1–20. 10.3389/fphys.2018.01294 30294279 10.3389/fphys.2018.01294 PMC6159745

[CR39] Martin L, Senesse P, Gioulbasanis I, Antoun S, Bozzetti F, Deans C, Strasser F et al (2015) Diagnostic criteria for the classification of cancer-associated weight loss. J Clin Oncol 33(1):90–99. 10.1200/JCO.2014.56.1894 25422490 10.1200/JCO.2014.56.1894

[CR40] Miyamoto Y, Hanna DL, Zhang W, Baba H, Lenz HJ (2016) Molecular pathways: Cachexia signaling - a targeted approach to cancer treatment. Clin Cancer Res 22(16):3999–4004. 10.1158/1078-0432.CCR-16-0495LK- 27340276 10.1158/1078-0432.CCR-16-0495 PMC4987228

[CR41] Mondello P, Lacquaniti A, Mondello S, Bolignano D, Pitini V, Aloisi C, Buemi M (2014) Emerging markers of cachexia predict survival in cancer patients. BMC Cancer 14(1):1–10. 10.1186/1471-2407-14-828 25400234 10.1186/1471-2407-14-828 PMC4239407

[CR42] Montalvo R, Counts BR, Carson JA (2018) Understanding sex differences in the regulation of cancer-induced muscle wasting. Curr Opin Support Palliat Care 12(August):1. 10.1097/SPC.0000000000000380 30102621 10.1097/SPC.0000000000000380 PMC6239206

[CR43] Ohmori H (2019) Evaluation of parameters for cancer-induced sarcopenia in patients autopsied after death from colorectal cancer. Pathobiology Ff 8521:306–314. 10.1159/000503037 10.1159/000503037 31707381

[CR44] Olaechea S, Sarver B, Liu A, Gilmore LA, Alvarez C, Iyengar P, Infante R (2023) Race, ethnicity, and socioeconomic factors as determinants of cachexia incidence and outcomes in a retrospective cohort of patients with gastrointestinal tract cancer. JCO Oncol Pract 19(7):493–500. 10.1200/OP.22.00674 37099735 10.1200/OP.22.00674 PMC10337717

[CR45] Ozorio GA, Barão K, Forones NM (2017) Cachexia stage, patient-generated subjective global assessment, phase angle, and handgrip strength in patients with gastrointestinal cancer. Nutr Cancer 69(5):772–779. 10.1080/01635581.2017.1321130 28524706 10.1080/01635581.2017.1321130

[CR46] Paczek S, Łukaszewicz-Zajac M, Gryko M, Mroczko P, Kulczyńska-Przybik A, Mroczko B (2020) CXCL-8 in preoperative colorectal cancer patients: Significance for diagnosis and cancer progression. Int J Mol Sci 21(6):1–13. 10.3390/ijms21062040 10.3390/ijms21062040 PMC7139325 32192002

[CR47] Peixoto S, Santos JMO, Silva MP, da Costa RM, Medeiros R (2020) Cancer cachexia and Its pathophysiology: links with sarcopenia, anorexia and asthenia. J Cachexia Sarcopenia Muscle 11(March):619–635. 10.1002/jcsm.12528 32142217 10.1002/jcsm.12528 PMC7296264

[CR48] Porporato PE (2016) Understanding cachexia as a cancer metabolism syndrome. Oncogenesis 5(2):1–10. 10.1038/oncsis.2016.3 10.1038/oncsis.2016.3 PMC5154342 26900952

[CR49] Prado CM, Sawyer MB, Ghosh S, Lieffers JR, Esfandiari N, Antoun S, Baracos VE (2013) Central tenet of cancer cachexia therapy: Do patients with advanced cancer have exploitable anabolic potential? Am J Clin Nutr 98(4):1012–1019. 10.3945/ajcn.113.060228 23966429 10.3945/ajcn.113.060228

[CR50] Ryan AM, Power DG, Daly L, Cushen SJ, Bhuachalla EN, Prado CM (2016) Cancer-asociated malnutrition, cachexia and sarcopenia: the skeleton in the hospital closet 40 years later. Proc Nutr Soc 75(2):199–211. 10.1017/S002966511500419X 26786393 10.1017/S002966511500419X

[CR51] Scheede-Bergdahl C, Watt HL, Trutschnigg B, Kilgour RD, Haggarty A, Lucar E, Vigano A (2012) Is IL-6 the best pro-Inflammatory biomarker of clinical outcomes of cancer cachexia? Clin Nutr 31(1):85–88. 10.1016/j.clnu.2011.07.010 21855185 10.1016/j.clnu.2011.07.010

[CR52] Srdic D, Plestina S, Sverko-Peternac A, Nikolac N, Simundic AM, Samarzija M (2016) Cancer cachexia, sarcopenia and biochemical markers in patients with advanced non-small cell lung cancer—chemotherapy toxicity and prognostic value. Support Care Cancer 24(11):4495–4502. 10.1007/s00520-016-3287-y 27236439 10.1007/s00520-016-3287-y

[CR53] Suetta C, Haddock B, Alcazar J, Noerst T, Hansen OM, Ludvig H, Kamper RS, Schnohr P, Prescott E et al (2019) The Copenhagen sarcopenia study: Lean mass, strength, power, and physical function in a Danish cohort aged 20–93 years. J Cachexia Sarcopenia Muscle 10(6):1316–1329. 10.1002/jcsm.12477 31419087 10.1002/jcsm.12477 PMC6903448

[CR54] Thålin C, Lundström S, Seignez C, Daleskog M, Lundström A, Henriksson P, Helleday T, Phillipson M, Wallén H, Demers M (2018) Citrullinated histone H3 as a novel prognostic blood marker in patients with advanced cancer”. PLoS ONE 13(1):1–17. 10.1371/journal.pone.0191231 10.1371/journal.pone.0191231 PMC5764486 29324871

[CR55] Thålin C, Aguilera K, Hall NW, Marunde MR, Burg JM, Rosell A, Daleskog M et al (2020) Quantification of citrullinated histones: development of an improved assay to reliably quantify nucleosomal H3Cit in human plasma. J Thromb Haemost 18(10):2732–2743. 10.1111/jth.15003 32654410 10.1111/jth.15003 PMC8722705

[CR56] Wang B, Thapa S, Zhou T, Liu H, Li L (2020) Cancer-related fatigue and biochemical parameters among cancer patients with different stages of sarcopenia. Support Care in Cancer 28:581–588. 10.1007/s00520-019-04717-0 10.1007/s00520-019-04717-0 31102055

[CR57] WHO (2019) Global Database on Body Mass Index. http://www.assessmentpsychology.com/icbmi.htm. Accessed 11 Sep 2019

[CR58] Wu L, Saxena S, Singh R (2020) Neutrophils in the tumor microenvironment. Adv Exp Med Biol 1224:1–20. 10.1007/978-3-030-35723-8 32036601 10.1007/978-3-030-35723-8_1 PMC7325741

[CR59] Yang R, Chang Q, Meng X, Gao N, Wang W (2018) Prognostic value of systemic immune inflammation index in cancer: a meta-analysis. J Cancer 9(18):3295–3302. 10.7150/jca.25691 30271489 10.7150/jca.25691 PMC6160683

[CR60] Zhou T, Wang B, Liu H, Yang K, Thapa S, Zhang H, Li L, Yu S (2018) Development and validation of a clinically applicable score to classify cachexia stages in advanced cancer patients. J Cachexia Sarcopenia Muscle 9(2):306–314. 10.1002/jcsm.12275 29372594 10.1002/jcsm.12275 PMC5879986

